# Novel mutations of *CHST6* in Iranian patients with macular corneal dystrophy

**Published:** 2009-02-18

**Authors:** Shiva Akbari Birgani, Zivar Salehi, Masoud Houshmand, Mohamad Javad Mohamadi, Leila Azizade Promehr, Zahra Mozafarzadeh

**Affiliations:** 1 Department of Biology, Guilan University of Medical Science, Rasht, Iran; 2 National Research Center for Genetic Engineering and Biotechnology, Tehran Iran

## Abstract

**Purpose:**

To characterize mutations within the carbohydrate sulfotransferase 6 (*CHST6*) gene in Iranian subjects from 12 families with macular corneal dystrophy (MCD).

**Methods:**

Genomic DNA was extracted from peripheral blood of 20 affected patients and 60 healthy volunteers followed by polymerase chain reaction (PCR) and direct sequencing of the *CHST6* coding region. The observed nucleotide sequences were then compared with those found by investigators in other populations with MCD and in the controls.

**Results:**

Analysis of *CHST6* revealed 11 different mutations. These mutations were comprised of six novel missense mutations (p.F55L, p.P132L, p.S136G, p.C149Y, p.D203Y, and p.H249R), one novel nonsense mutation (p.S48X), one novel frame shift (after P297), and three previously reported missense mutations (p.P31L, p.C165Y, and p.R127C). The majority of the detected MCD mutations are located in the binding sites or the binding pocket, except the p.P31L and p.H249R mutations.

**Conclusions:**

Nucleotide changes within the coding region of *CHST6* are predicted to significantly alter the encoded sulfotransferase within the evolutionary conserved sequences. Our findings show that *CHST6* mutations are responsible for the pathogenesis of MCD in Iranian patients. Moreover, the observation that some cases of MCD cannot be explained by mutations in the coding region of *CHST6* suggests that MCD may result from possible upstream rearrangements in the *CHST6* genomic region.

## Introduction

Corneal dystrophies are a heterogeneous group of disorders that may lead to severe visual impairment of which macular corneal dystrophy (MCD; OMIM 217800) is transmitted as an autosomal recessive trait [1]. The prevalence of MCD varies immensely in different parts of the world, but in most populations, the condition is rare. As a treatment, penetrating keratoplasty is performed when useful vision has been lost. Reportedly, in Iceland and Japan, MCD accounts for 75% and 10%, respectively, of the corneal dystrophies requiring corneal grafting [2,3]. Clinical symptoms usually manifest within the first decade of life, and MCD patients show spotted opacity in both corneas. These abnormal deposits are associated with a central stromal haze that gradually extends to the periphery of the cornea, leading to visual impairment, and patients require keratoplasty [4]. MCD has been subdivided into three immunophenotypes (MCD types I, IA, and II) based on the reactivity of the patient's serum and corneal tissue to monoclonal anti-keratan sulfate antibody (5D4 anti-KS antibody) [5].

Biochemical studies have indicated that a specific sulfation step of keratan sulfate is impaired in MCD, resulting in an accumulation of glycosaminoglycans (GAGs). Keratan sulfate proteoglycan, the most abundant carbohydrate in the cornea, plays an important role in maintenance of corneal transparency [6,7]. Keratan sulfate consists of a linear poly-*N*-acetyllactosamine chain that carries sulfate residues on C-6 of *N*-acetylglucosamine (GlcNAc) and galactose (Gal). Because the sulfation of carbohydrates affects their biochemical characteristics such as water solubility and electrical charge, this modification appears to be important for the function of keratan sulfate proteoglycans in the cornea. Considering the importance of the sulfated carbohydrates of keratan sulfate in the normal function of the cornea, it has been suggested that lack of this sulfation step is a major cause of MCD [6,8,9].

It has also recently been shown that there is a failure in the activity of corneal GlcNAc 6-*O*-sulfotransferase (C-GlcNAc6ST), which transfers sulfate to position 6 of GlcNAc residues and this enzyme participates in biosynthesis of corneal keratan sulfate proteoglycan. In the cornea of patients with MCD, a decrease in C-GlcNAc6ST activity results in the formation of poorly sulfated or non-sulfated keratan sulfate and causes corneal opacity [10,11].

Using genetic linkage analysis, the critical region for MCD has been mapped to chromosome 16 (16q22). The carbohydrate sulfotransferase 6 gene (*CHST6*) was identified within the MCD critical region encoded C-GlcNAc6ST, and mutations in *CHST6* have been shown to be the fundamental defect in MCD [12,13].

In the present study, which is the first comprehensive work studying Iranian subjects with MCD, we screened 20 patients with MCD from 12 families to search for mutations in *CHST6*. We identified seven novel mutations and four previously reported disease-causing mutations.

## Methods

### Patients

This study was performed in accordance with the Declaration of Helsinki and with the approval of the ethics board of the Amiralmomenin Hospital and Guilan University (Rasht, Iran). Twenty patients from 12 unrelated Iranian families who received clinical diagnosis of MCD were included in this study. Diagnosis for MCD in affected individuals was made by cornea specialists at the Amiralmomenin Hospital (associated with Guilan University of Medical Sciences). All patients were older than 25 years, and the obtained pedigrees were consistent with an autosomal recessive inheritance pattern. There was not any known consanguinity in the patients' families. Sixty unrelated, unaffected, and healthy volunteers were recruited to serve as controls.

Blood samples were obtained from a peripheral blood vessel from affected patients and healthy volunteers. Genomic DNA was extracted by standard procedure using the PAX gene blood DNA kit (Qiagen, Valencia, CA).

### DNA analysis

The coding region of *CHST6* was amplified using polymerase chain reaction (PCR) and the primers designed to create three overlapping amplicons. The oligonucleotide primers used were identical to those reported by Akama and associates [12].

Each reaction was performed in a 50-µl volume comprising of genomic DNA (100 ng), 10X PCR buffer, 0.2 µM dNTP, 1.5–2 µM of MgCl_2_, 0.2 µM of each primer, 1 U of Taq DNA polymerase (Qiagen). The thermal cycling was performed using the following protocol 2 min at 96 °C and 35 cycles of 30 s at 96 °C, 30 s at 57 °C, 45 s at 72 °C, and final extension 7 min at 72 °C followed by a 4 °C hold cycle.

The PCR products were then purified using the QIAquick PCR purification kit (Qiagen) and sequenced on both strands by the ABI BigDye terminator chemistry and an ABI Prism 3700 instrument (Applied Biosystems, Foster City, CA). Sequences were analyzed using the Sequencher software (Gene Codes Corporation, Ann Arbor, MI) and compared with the nucleotide sequence of the *CHST6* human cDNA (GenBank accession number AF219990).

## Results

A total of 20 affected patients representing 12 different families were enrolled in the study. The most abnormalities found in this study were single base changes in the coding region of *CHST6* that altered a coded amino acid (Table 1), seven of which were homozygous. The nucleotide changes included c.92C>T, c.143C>A, c.165C>A, c.379C>T, c.406A>G, and c.746A>G, resulting in a proline to a leucine substitution (p.P31L), a serine to stop codon substitution (p.S48X), a phenylalanine to a leucine substitution (p.F55L), an arginine to a cysteine substitution (p.R127C), a serine to a glycine (p.S136G), and a histidine to an arginine substitution (p.H249R).

**Table 1:** *CHST6* mutations in Iranian patients with MCD.

**Family**	**Number of patients**	**Zygosity**	**Nucleotide change**	**Protein change**	**Disease-causing or not**
A	3	Homozygous	c.746A>G	p.H249R	+
B	3	Homozygous	c.92C>T	p.P31L	+
C	2	Homozygous	c.891–892insC	p.P297fs	+
D	1	Homozygous	c.143C>A	p.S48X	+
E	4	Heterozygous	c.494G>A	p.C165Y	+
		Heterozygous	c.395C>T	p.P132L	+
F	1	Heterozygous	c.446G>A	p.C149Y	+
G	1	Homozygous	c.379C>T	p.R127C	+
H	1	Heterozygous	c.607G>T	p.D203Y	+
I	1	Homozygous	c.406A>G	p.S136G	+
J	1	Homozygous	c.165C>A	p.F55L	+
K	1	Heterozygous	c.405C>T	-	-
L	1	No mutations	-	-	-

In the table “+” denotes that the observed changes are Disease-causing, and “-” denotes that the observed changes are not Disease-causing.

In family C, an insertion of a single base pair between nucleotides 891 and 892 was identified, resulting in a frame shift after codon, P297 (p.P297fs).

In addition, a compound heterozygosity was detected in family E. In this family, a heterozygous changes, c.395C>T and c.494G>A, was identified, predicting amino acid changes of proline to leucine (p.P132L) and cysteine to tyrosine (p.C165Y).

In two families, only one heterozygous pathogenic change was observed in the coding *CHST6* sequence. In family F, we found c.446G>A, which replaces a cysteine to a tyrosine (p.C149Y). In family H, c.607G>T was found, replacing an aspartic acid to a tyrosine (p.D203Y).  In the last two families (K and L), no presumable pathogenic change was detected. In addition, a single novel nucleotide polymorphism, c.405C>T (a heterozygous change), was found in family K. It was considered a non-pathogenic sequence variant because it had no effect on the amino acid.

The amplified PCR products from 60 control individuals were analyzed for each alteration using direct sequencing, none of which indicated the mutations seen for the patients.

## Discussion

In this study, we examined the *CHST6* coding region for mutations in 20 individuals with MCD from 12 Iranian families. Sequencing analysis revealed six novel missense mutations (p.F55L, p.P132L, p.S136G, p.C149Y, p.D203Y, and p.H249R), one nonsense mutation (p.S48X), one frame shift (p.P297fs), and three previously reported missense mutations (p.P31L [13], p.C165Y [14], and p.R127C [15]) in *CHST6* in the Iranian patients with MCD.

**Figure 1:**
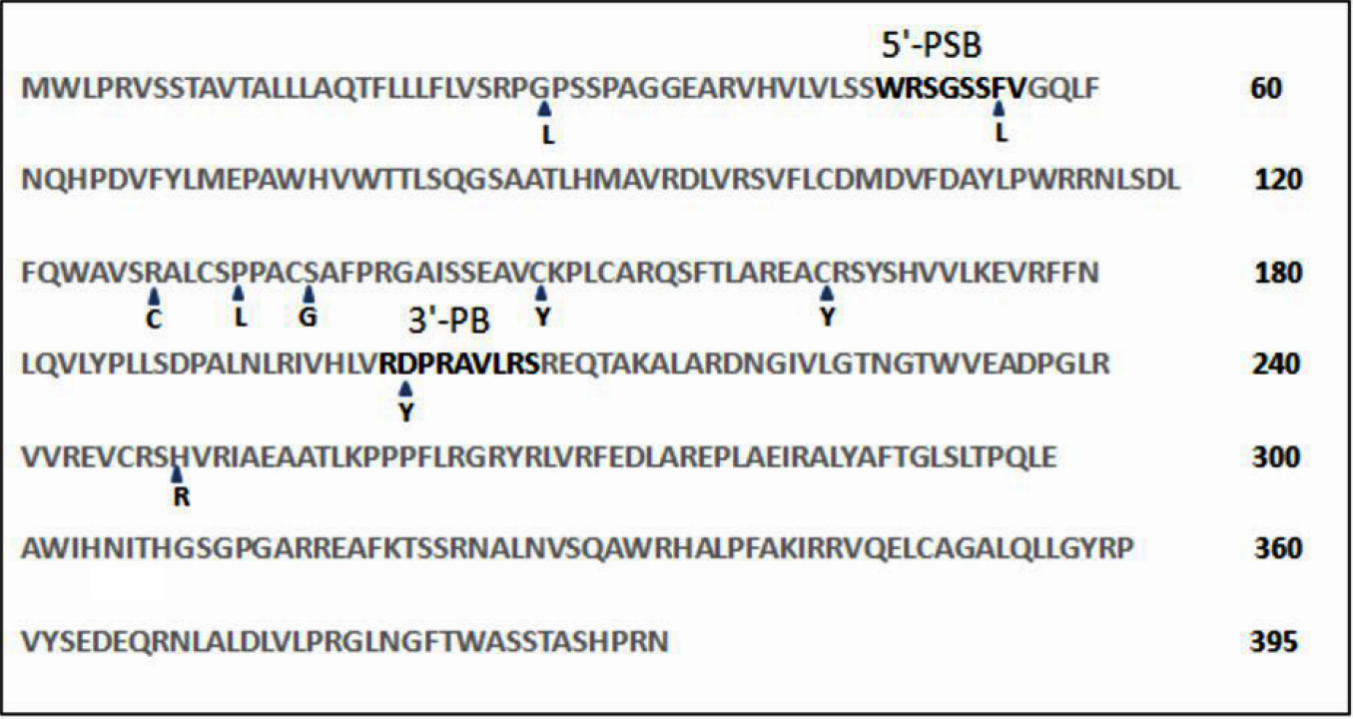
Amino acid sequence of C-GlcNAc6ST. This diagram shows the location of missense mutations in Iranian patients with MCD. These mutations are marked by amino acid position using letters with blue arrowheads. Bold letters show the location of the 5′phosphosulfate binding site (5′-PSB) and the 3′ phosphate binding site (3′-PB).

*CHST6* encodes the C-GlcNAc6ST, comprising 395 amino acids. This enzyme is localized in the Golgi apparatus and is a member of the Gal/GalNAc/GlcNAc 6-*O* sulfotransferase (GST) family. The C-GlcNAc6ST contains a short cytosolic tail at the NH_2_-terminal, a single transmembrane span, and a COOH-terminal domain that contains two putative binding sites for the high energy sulfate donor (adenosine3′-phosphate-5′phosphosulfonate [PAPS]). One of the binding sites is the 5′-phosphosulfate binding site (5′-PSB), corresponding to amino acid 49–56, and the other is the RX_7_S 3′-phosphate binding site (3′-PB), corresponding to amino acid 202–210 (Figure 1). The sequence between the two binding sites is thought to contribute to a binding pocket that interacts with an acceptor (GlcNAc) to bring it into apposition with the sulfate donor [16].

It is reported that mutations of these binding motif sequences abolish the sulfotransferase activity in the case of other glycosaminoglycan sulfotransferases, indicating important catalytic roles of these amino acid residues [17].

The identified mutation of p.F55L is located in the 5'-PSB domain. The mutations of p.R127C, p.P132L, p.S136G, p.C149Y, p.C165Y are located in the sequence between the 5'-PSB domain, and the 3'-PB domain. The mutation of p.D203Y is located in the RX_7_S sequence for the 3'-PB domain. Whereas the mutations of p.P31L, p.H249R are neither located in the binding motifs nor in the binding pocket.

Amino acid changes at positions 203 and 249 have been previously reported by Akama et al. [12] and Warren et al. [18], respectively, but they are different from the mutations identified in the present study. We have observed c.607G>T causing a replacement of an aspartic acid to a tyrosine (p.D203Y) while in Japanese patients with MCD, a change at nucleotide position 609 (c.609C>A) causes the replacement of an aspartic acid to a glutamic acid at the same protein position (p.D203E) [12]. In Iranian subjects, a change at nucleotide position 746 (c.746A>T) leads to the replacement of a histidine to an arginine at position 249 of the protein (p.H249R) while in Indian patients with MCD, Warren et al. [18] reported c.746A>C resulting in the replacement of a histidine to a cysteine (p.H249C). The identified nonsense coding region mutation would clearly have a significant impact on the encoded enzyme. The nonsense mutation (p.S48X) would result in a premature termination of the encoded protein. Likewise, the insertion of one nucleotide (c.891–892insC) will cause a reading frame shift. It is noteworthy that the occurrence of frame shift and nonsense mutations may lead to the elimination of the protein due to nonsense-mediated decay of the mRNA (NMD). NMD in mammalian cells generally degrades mRNAs that terminate translation more than 50–55 nucleotides upstream of a splicing-generated exon-exon junction [19,20]. NMD downregulates spliced mRNAs that prematurely terminate translation so production of the potentially toxic truncated proteins that they encode does not occur [21].

It is notable that previously only one patient of Iranian origin with MCD was studied. Deletion of the whole open reading frame was identified as the cause in this individual [22]. Additional evidence to support our findings that the sequence changes identified are responsible for producing inactivation of the *CHST6* gene product is provided by the fact that no mutations were detected in the *CHST6* coding region from control individuals.

In one family (family L), there were no coding region mutations, and in another family (family K), the nucleotide change had no effect on protein phenotype. These cases of MCD can not be explained by mutations in the coding sequence of *CHST6*, suggesting that MCD may result from possible upstream rearrangements such as the promotor, which is also part of *CHST6* and has not been analyzed in the present study. It is intriguing that such a large number and wide variety of novel mutations were discovered throughout the *CHST6* coding sequence. This suggests that in contrast to *TGFBI* (transforming growth factor, beta induced) with its hot spots for corneal dystrophy-associated amino acid substitution, *CHST6 *does not demonstrate a highly conserved number of disease-causing mutations [23].

In summary, we identified seven novel and three previously reported *CHST6* mutations in our panel consisting of 20 Iranian MCD patients from 12 families. Further analysis of these mutations may help to achieve a clearer understanding of the molecular events leading to the characteristic lesions of MCD.
